# Nuclear pore complex plasticity during developmental process as revealed by super-resolution microscopy

**DOI:** 10.1038/s41598-017-15433-2

**Published:** 2017-11-07

**Authors:** Julien Sellés, May Penrad-Mobayed, Cyndélia Guillaume, Alica Fuger, Loïc Auvray, Orestis Faklaris, Fabien Montel

**Affiliations:** 1 Matière et Systèmes Complexes, Université Paris Diderot/CNRS (UMR 7057), 75205 Paris, Cedex 13 France; 2 Institut Jacques Monod, Université Paris Diderot/CNRS, UMR 7592, 15 rue Hélène Brion, 75205 Paris, CEDEX 13 France; 3 ImagoSeine core facility, Institut Jacques Monod, Université Paris Diderot/CNRS, UMR 7592, 15 rue Hélène Brion, 75205 Paris, CEDEX 13 France; 4 Univ Lyon, Ens de Lyon, Univ Claude Bernard, CNRS, Laboratoire de Physique, F-69342 Lyon, France

## Abstract

Nuclear Pore Complex (NPC) is of paramount importance for cellular processes since it is the unique gateway for molecular exchange through the nucleus. Unraveling the modifications of the NPC structure in response to physiological cues, also called nuclear pore plasticity, is key to the understanding of the selectivity of this molecular machinery. As a step towards this goal, we use the optical super-resolution microscopy method called direct Stochastic Optical Reconstruction Microscopy (*d*STORM), to analyze oocyte development impact on the internal structure and large-scale organization of the NPC. Staining of the FG-Nups proteins and the gp210 proteins allowed us to pinpoint a decrease of the global diameter by measuring the mean diameter of the central channel and the luminal ring of the NPC via autocorrelation image processing. Moreover, by using an angular and radial density function we show that development of the *Xenopus laevis* oocyte is correlated with a progressive decrease of the density of NPC and an ordering on a square lattice.

## Introduction

The unique gateway for bidirectional molecular exchange between the nucleus and the cytoplasm of eukaryotic cells is a complex molecular machinery spread across the nuclear envelope and called Nuclear Pore Complex (NPC)^1–4^. Whereas its composition and size may vary between different species, its general structure and function appear to be strikingly conserved^5,6^. The NPC is a large 60 to 125 MDa complex, composed of at least 30 different proteins, termed nucleoporins which are each present in multiple copies^7,8^. Recent studies have shown that half of these proteins are involved in the scaffolding of the NPC and arranged in an eight-fold symmetrical structure, such as the gp210 proteins, whereas the rest of the proteins, among which are the so-called FG-Nups, are considered to be unstructured and highly fluctuating^9,10^. Due to its size (>100 nm), its complexity and its internal dynamics, the study of NPC remains an experimental and theoretical challenge.

In the past years new approaches have been developed to achieve a better understanding of the NPC structure in response to external cues. Integrative approaches combining electron microscopy, proteomics and other biophysical tools have led to localize most of the constituents of the complex^10–12^. Cryo-electron microscopy in conjunction with subtomogram averaging have produced a 3D structure with a resolution of the NPC scaffold approaching 2 nm^9^. More recently, optical super-resolution microscopy has also been used successfully to study the structure of the NPC with a high molecular specificity of the labeling under physiological conditions^13–16^. In particular the ring shape eight fold symmetry of the pore has been resolved^17^ and the relative positions of the components of the Y complex, which is the essential building block of the NPC, have been measured^18^.

The modifications of the nuclear pore structure, also called nuclear plasticity, have been assessed using electron microscopy^19–25^. Cryo-electron microscopy studies have shown the existence of natural variants and modifications of its structure^9,26,27^, while atomic force microscopy has demonstrated a reversible mechanical gating of the nuclear pore^28–31^.

Using *d*STORM super-resolution microscopy^32,33^, we showed that *Xenopus Laevis* oocyte development impacts on the structure and the large scale organization of the NPCs. By following some relevant parameters during developmental process, such as the internal and external nuclear pores diameter or their organization, we highlight structural modifications of the NPCs.

## Results and Discussions

### dSTORM imaging of the NPCs of Xenopus oocyte nuclear envelope

**Figure 1:**
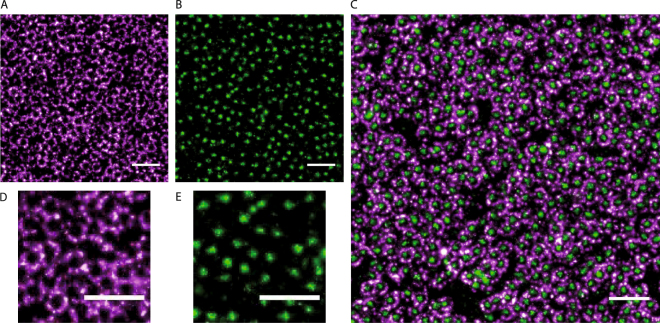
*d*STORM imaging of the nuclear envelopes from *X. laevis* oocytes. (**A**) *d*STORM image of a spread nuclear envelope labelled with anti-gp210 primary antibody and Alexa647 secondary antibody. (**B**) *d*STORM image of a spread nuclear envelope labelled with WGA-Alexa488. Scale bar 500 nm. (**C**) *d*STORM image of a spread nuclear envelope labelled with WGA-Alexa488 (green) and gp210-Alexa647 (magenta). (**D**) Zoom on the image (A). **(E)** Zoom on the image (B).

**Figure 2:**
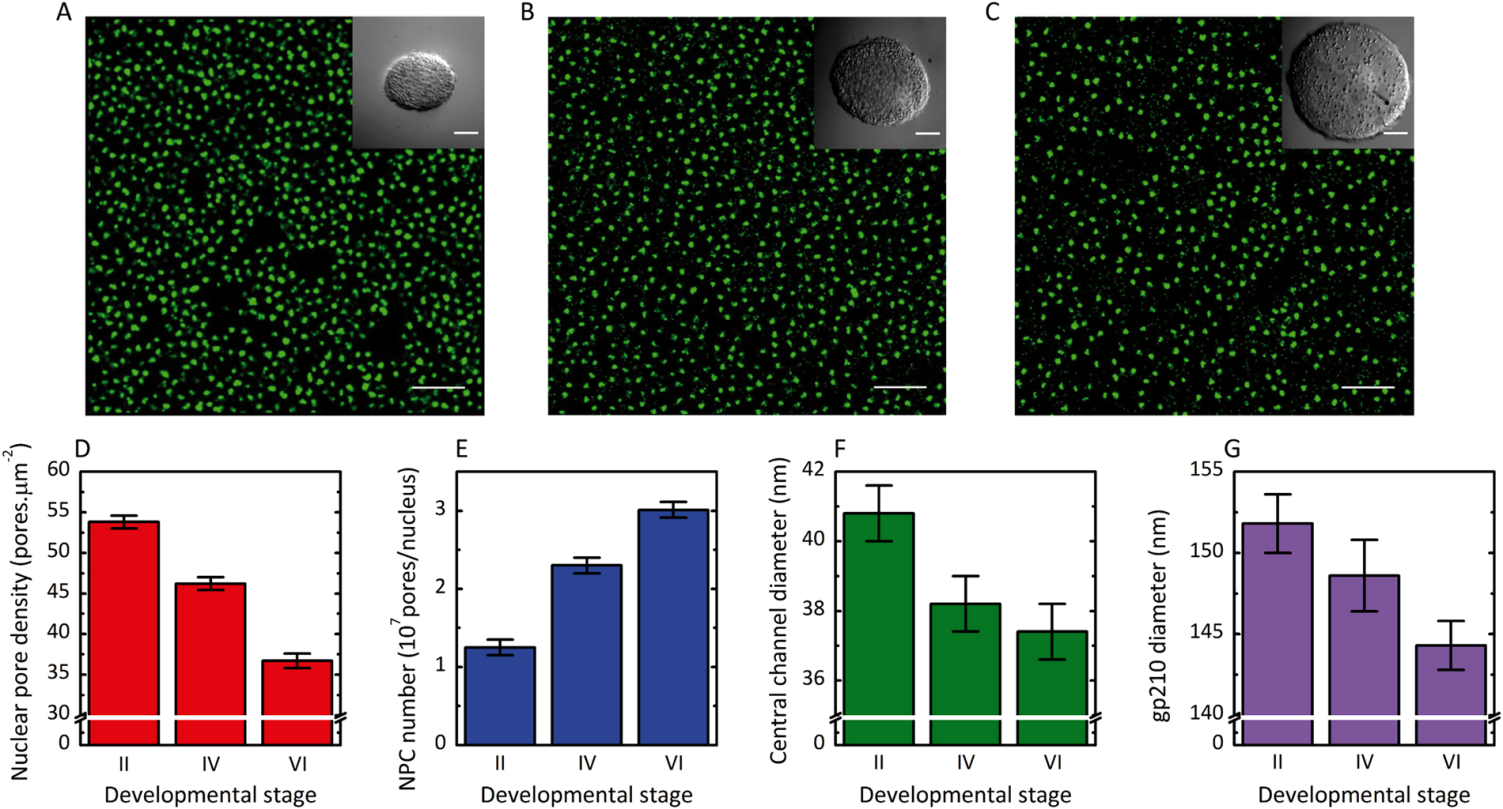
Effect of oocyte development on the density, the number and the diameter of the nuclear pore complexes. (**A**–**C**) *d*STORM images of nuclear envelopes from oocytes respectively at stage II, IV and VI. The central channel is labelled with fluorescent WGA-Alexa647. Scale bar 5 µm. Insets: Stereomicroscope images of the oocyte respectively at stage II, IV and VI. Scale bar 50 µm. (**D**) Effect of oocyte development on nuclear pore complex density. (**E**) Effect of oocyte development on nuclear pore number per nucleus. (**F**) Effect of oocyte development on central channel diameter. (**G**) Effect of oocyte development on gp210 diameter. For each condition, the number of investigated NPC is superior to 300 000. Errors are experimental standard errors. The precision of the measurements was assessed by bootstrapping and by comparing different rounds of experiments.

**Figure 3:**
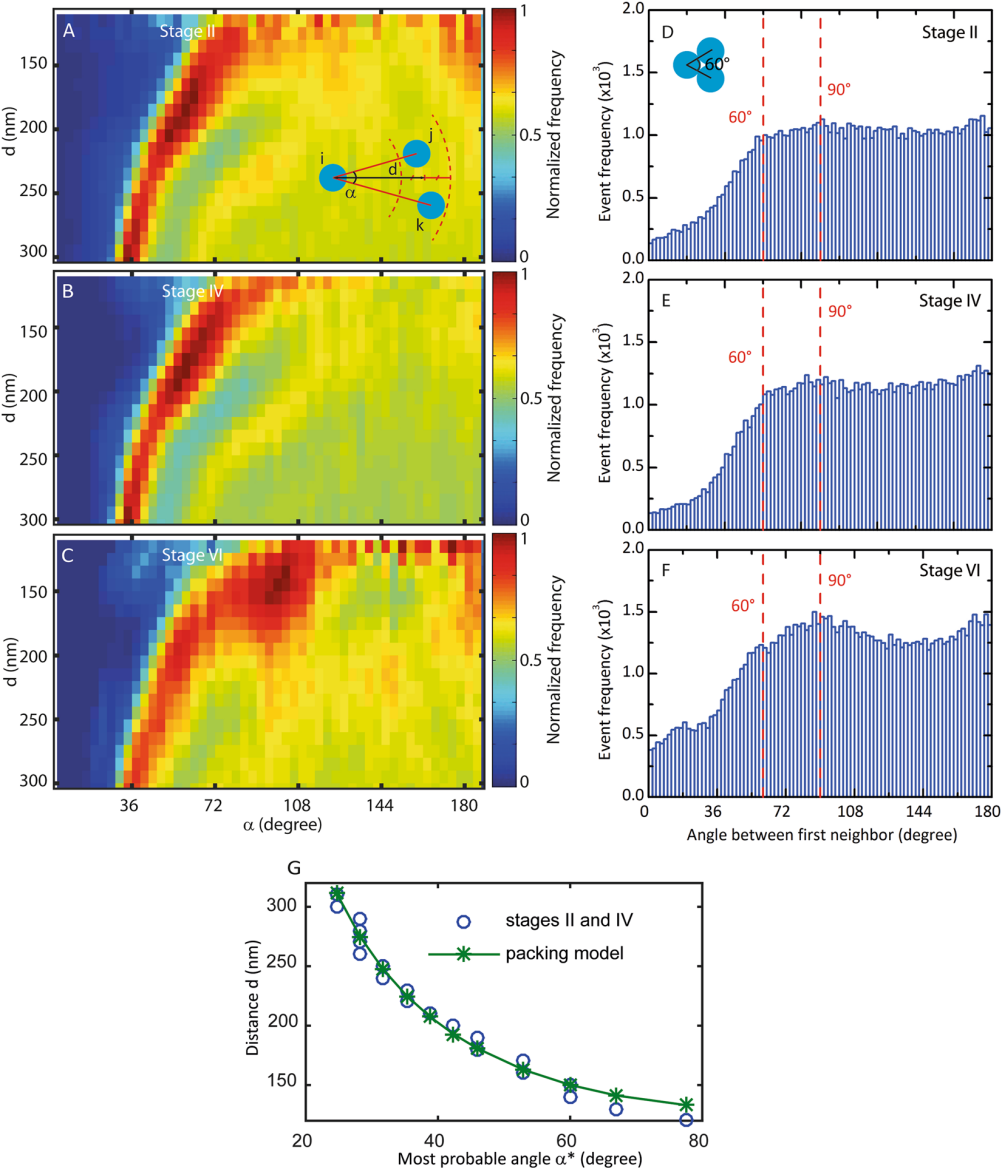
Effect of oocytes development on the organization of the nuclear pore complexes. (**A**–**C**) 2D histogram of the probability P(d,α) to observe a NPC on a given envelope with two neighbors at a distance d and forming an angle α respectively for oocytes at stage II, IV and VI. For stage VI oocytes the most probable coordinates are (135 ± 5 nm, 90 ± 6°). The frequency event is normalized by the maximum for each histogram. **(D**–**F)** First neighbor angle distribution evolution for respectively stage II, stage IV and stage VI oocytes. The red dashed line at 60° corresponds to the minimal angle possible between 3 NPCs. (**G**) Experimental most probable angle α^*^ between three nuclear pore complexes as a function of the distance d between the central pore and its two neighbors (dashed line) and theoretical angle α between three nuclear pore complexes as a function of the distance d between the central pore and its two neighbors in the packing model (solid line). In this model all the nuclear pore complexes are in contact with their first neighbors but without any large-scale order.

We have used *d*STORM super-resolution imaging to gain insight for the first time into the organization and structure of the NPCs during oocytes development of *X. Laevis*. Xenopus nuclear envelopes were extracted and labelled at different stages of oocyte development using a protocol modified from earlier studies^34,35^ in order to preserve the integrity of the membrane and to ensure adhesion of the envelope to the glass coverslip (see Materials and Methods). Membranes were labeled using either a fluorescent wheat germ agglutinin (WGA-Alexa647 or WGA-Alexa488), which has a high affinity for N-acetylglucosamine modifications of the nucleoporins present in the nuclear pore central channel^36,37^, and/or a primary antibody (AB) against the gp210 protein coupled to a secondary mouse AB with a fluorescent marker (Alexa647). Ultimate structure of the NPCs was visualized using a *d*STORM microscope (Zeiss Elyra P.S.1) (Fig. 1). At least 8 samples were imaged for each condition and more than 3 images were acquired for each sample. Each reconstructed image encompassed the recording of more than 30 000 frames, with at least 1 000 000 discrete localizations per sequence. Typically, around 30 000 NPCs were visible in each image. Thus, the analysis of more than 300 000 nuclear pores for each condition enabled us to uncover very subtle changes in the measured parameters. On the reconstructed image, the center of mass for each individual nuclear pore central channel was automatically detected. The diameters of the NPC central channel and the luminal ring (Fig. 2F,G) as well as the NPC density (Fig. 2D) were calculated and near neighbor angles were measured (Fig. 3D–F). The average image of the nuclear pore central channel was computed by combining individual reconstructed images of the pores (N = 270 000). In order to check whether the WGA labelling was not overestimating the measured densities of nuclear pores, the membrane was double stained with labeled WGA and anti-gp210 antibody (Fig. 1B). The NPCs density was similar in both cases, the percentage of pores labeled by WGA only being less than 6%.

### Effect of oocyte development on the density, number and diameter of the NPCs

Despite the fact that *X. Laevis* oocyte is one of the favorite model for the study of NPCs (see^6^ for a review), only two studies were carried out on early stage oocytes using electron microscopy^38^ or Atomic Force Microscopy (AFM)^28^. The other investigations involve late stage of oogenesis, i.e. stage VI^39^, because they are easier to handle due to their large size. As shown in Fig. 2D, the early stage II exhibit the highest density of NPCs with 53.8 ± 0.9 NPC.µm^−2^, while it was 46.2 ± 0.9 NPC.µm^−2^ at stage IV and 36.7 ± 0.8 NPC.µm^−2^ at stage VI (errors are experimental standard errors). The total number of NPCs per nucleus was computed from the diameter of the nuclei and nuclear pore density (Fig. 2E). Stage II oocytes had a lower number of NPCs (1.25 × 10^7^) than stage IV oocytes (2.3 × 10^7^) and stage VI oocytes (3.01 × 10^7^). These results corroborate recent measurements carried out on native nuclear membranes from late stage oocytes by atomic force microscopy^28^ and super-resolution microscopy^17^ where the density of nuclear pores and the total number of NPCs per nucleus are in agreement with our observations. In contrast, these figures differ from the measurements obtained previously using negative staining methods and EM^38^. These discrepancies can be attributed to differences in sample preparation as discussed by Schlune *et al*.^28^ and come from the negative staining method which tends to select nuclear envelopes fragments with the highest pore frequency.

One of the major finding of our study is the observation of a decrease of the central channel and the scaffold diameter of the NPCs. Using optical super-resolution microscopy, which is compatible with specific labelling, we show a new structural plasticity of the NPCs at the level of the FG-Nups present in the central channel and at the level of the gp210 proteins involved in the scaffold of the NPCs. The NPCs of stage II oocytes had a central channel diameter of 40.8 ± 0.8 nm, at stage IV a diameter of 38.2 ± 0.8 nm (p(II-IV) < 0.01) and a diameter of 37.4 ± 0.8 nm at stage VI (p(II-VI) < 0.01). We can notice that the size of the central channel at stage VI is similar to that reported by optical super-resolution microscopy by Löschberger *et al*.^17^. In our case, the majority of the central channels of the NPCs is a compact structure without hole. This observation does not depend on the reconstruction parameters or the labelling protocol of the envelope (Figs S1 and S2). A similar compact structure has also been reported by STED super-resolution microscopy by Göttfert *et al*.^14^. For the luminal ring, we measure a diameter of 151.8 ± 1.8 nm for stage II oocytes, 148.6 ± 2.2 nm (p(II-IV) < 0.01) for stage IV and 144.3 ± 1.5 nm (p(II-VI) < 0.01) for stage VI (errors are experimental standard errors). Precision of all measurements was assessed by bootstrapping and by comparing different rounds of experiments^40^. Since previous studies show that transcriptional activity drops very significantly from a high level at early stage to an almost undetectable level at the late stage^28,41^, we argue that the dilatation may be linked to a direct mechanical effect such as the increase of the flow of matter like RNA circulating through the NPCs. Another explanation could be an allosteric switch in the structure of the pore as already observed for some of the FG-Nups present in the nuclear pore complex^42^ or other types of neuronal channels^43–45^. A separate study would be necessary to understand the physiological mechanism of this effect.

### Effect of development stage on the lattice structure

The large-scale organization of the NPCs on the nuclear envelopes for stages II, IV and VI was determined by calculating the angle distribution between first neighbors for each NPCs and by using the angular and radial density function P(d,α). The latter describes the probability to observe on a given envelope a NPC with two neighbors at a distance *d* and forming an angle α. Qualitatively, we observed that the NPCs in earlier stages (II and IV) appeared to be less organized than in later stages (VI) as is it visible in Fig. 2A–C. Stage VI nuclear envelopes displayed small clusters of nuclear pores organized in square lattice with typical lateral size of 3 to 4 pores. In order to quantify this effect we measured the angle distribution function between the first two neighbors of a given NPC (Fig. 3D–F). For stage II and stage IV oocytes we can see a flat distribution which means that there are no preferential angles while at stage VI we have two distinct peaks at 90° and 180° (Fig. 3D–F). In order to investigate further this structure at all scales, different tools developed to analyze 2D and 3D crystal structure could be used such as higher order radial density function^46^ or bond-orientational order parameter^47–49^. We chose to use the angular and radial density function P(d,α) (Fig. 3A–C). This map can be seen as a measurement of the 2D order at all scales in the structure. A completely random structure will show a flat 2D map whereas a crystal-like structure will have a point like 2D map (Fig. S2). We observed two distinct behaviors for the early (II, IV) and late (VI) stages. In the case of stage II and stage IV we observed a map characteristic of a 2D dense amorphous structure. The maps could be divided into three regions: a low probability region corresponding to the close contact exclusion between discs of the same diameter, a high probability region corresponding to 2 neighbors in contact at a distance *d* and a flat region corresponding to non-overlapping NPC. In order to show that the high probability region corresponded to an amorphous organization of packed nuclear pores, we represented the most probable angle as a function of the distance between the NPC and its two neighbors. On the same axis we also represented the result of a simple packing model. In this model we assumed that the nuclear pores were closely packed. Then, the most probable angle α^*^ formed by three NPC was determined by the contact at a distance d of the two other complexes which gives: sin α^*^ = d_NPC_/d with d_NPC_ the diameter of the complex. We observed a clear agreement between this model without any free parameter and the experimental data (Fig. 3G). This result showed that the structure at the early stages could be indeed described as a compact and amorphous structure of nuclear pore complexes without any particular order. In the case of later stage oocytes, we detected, in addition to the regions previously observed for early stages, a new localized high probability peak (d = 135 +/− 5 nm, α = 90 +/− 6°). This peak indicates the presence of neighbors positioned at the first neighbor distance and organized on a square lattice. This result showed quantitatively that the structure observed for late stage oocyte could be described as a mixture of amorphous and square lattice domains. These calculations demonstrate that the pores become organized and gradually form nano-domains with a square lattice structure on the nuclear envelope in the course of development. The first indication for a square lattice order in the NPCs organization in *X. laevis* oocyte has been provided by Unwin *et al*.^38^ for stage VI. Here we directly show that this order appears actually during oocyte development. Because it is already detectable at a low NPCs density, we propose that it is mediated by the gradual production of a structural constituent of the nuclear envelope. Lamins, which interact with the nuclear basket of the pore^50–54^, could be involved in this process^55–57^ and the molecular mechanism leading to this organization deserves further investigation.

Overall, we report for the first time that the oocyte development impacts on the nuclear organization and the structure of NPCs. Our studies could pave the way towards extensive works on how the structure of NPCs are linked with physiological activity and on the relation between the large scale organization of NPCs and the constituent of the nuclear envelope.

## Materials and Methods

### Living material

*Xenopus laevis* were purchased from “UMS 3387 - Centre de Ressources Biologiques Xénopes” and raised at 18 °C standard conditions in Xenoplus-amphibia housing system (Techniplast), illuminated with a photoperiod of 12 hours, in the animal housing facility at the Jacques Monod Institute (licence number B-75-13-17). Ovarian biopsies were performed on adult females anesthetized in 0.15% Tricaine methane sulfonate (MS222, Sigma Chemical, St. Louis, MO). Oocytes of different stages were sorted according to their distinct morphological features^39^ and incubated for 24 h at 18 °C in MBS Buffer (Modified Barth’s Solution). All animal experiments were performed in accordance with the approved protocols and guidelines at Paris Diderot University by the Animal Experimentation Ethical Committee Buffon (CEEA-40) supervised by the French ministry for education and research.

### Nuclear envelope preparation and labelling

For *d*STORM imaging of the nuclear envelopes of Xenopus oocytes at different stages, we used a protocol that is modified from Peters *et al*.^35^, Löschberger *et al*.^17^ and Penrad-Mobayed *et al*.^34^ in order to preserve the integrity of the membrane and to ensure the adhesion of the envelope with the glass coverslip even for early stages oocytes. Oocytes were transferred in 3:1 medium (75 mM KCl, 25 mM NaCl, Tris-HCl, pH 7.2). The nucleus is isolated by gentle pipetting after dissecting the oocyte using two pairs of forceps (Dumont N°5). The yolk granules still adhering to the nuclear envelope is removed by gentle backs and forth movement of the nucleus in the pipette. The nucleus is then transferred into a micro-chamber, filled with the same medium. The micro-chamber consists of a well with a circular hole (2.5 mm of diameter) in a glass slide, at the bottom of which a cover slide (Zeiss type 1.5, high precision, thickness 170 ± 5 µm) is sealed with cyanoacrylate glue. To open the small nucleus at early oocyte stages (approximately 250 μm of diameter for stage II), we used insect pins of 0.1 mm of diameter (Austerlitz stainless steel insect pins 0.1 mm) instead of the Dumont forceps, largely used for IV-VI oocyte stages. Furthermore, in order to make the nuclear envelope firmly attached to the cover slide before subsequent treatments, the nucleus is pressed to the bottom of the micro-chamber using blunt-end glass capillary. After dissection, the preparation is centrifuged at 300 g for 15 min, at 4 °C. After centrifugation, the samples were fixed for 20 min with 2% paraformaldehyde in phosphate-buffered saline (PBS), twice washed with PBS, saturated with 0.5% bovine serum albumin (BSA) for 10 min, labelled with fluorescent wheat germ agglutinin (WGA-Alex647, 1 µg.ml^−1^, Thermo Fisher Scientific) which is a lectin with a high affinity for N-acetylglucosamine modifications of the nucleoporins present in the nuclear pore central channel^36,37^. For the labelling of gp210, a mouse monclonal antibody against Xenopus gp210 kindly supplied by George Krohne (Wuerzburg University) have been used with the modified staining protocol of Löschberger *et al*.^58^, by adding a 5 min permeabilization step with 0.5% Triton-X100 between the fixation and the saturation steps. The preparations can be stored in PBS at 4 °C for 24–48 h before *d*STORM imaging.

### dSTORM imaging

All the *d*STORM imaging was performed with a Zeiss Elyra P.S.1 microscope. In order to estimate the drift sample during imaging, we used either multicolor TetraSpeck beads (100 nm diameter, TetraSpeck; Life Technologies) as fiducial markers or either cross-correlation based algorithm from Zeiss-ZEN acquisition software. The photoswitching buffer was a commercial buffer from the Abbelight Company. The preparation was covered with a cover slide, sealed with a polymerized liquid (Twinsil Silicone, Rotec) and first observed under 10x air objective to look for the nuclear envelope. Then the region to be observed with *d*STORM was selected with the 100x, NA 1.46, Zeiss plan-APO objective. With laser illumination at 642 nm and an emission filter matched to A647 emission spectrum (LP 655), we acquired 30000 raw images of blinking molecules, under total internal reflection fluorescence (TIRF) microscopy mode. The camera used was an EMCCD Andor iXon 897 (pixel size 16 µm, use of an Optovar lens magnification 1.6x, so the objective 100x used is yielding a final pixel size of 100 nm). Fluorophore positions were computed using the Zeiss-Zen super-localization software (ZEN). Briefly, the determination of the x-y coordinates of the fluorophores is achieved by approximation of a two-dimensional Gaussian function to the fluorescence emission pattern of individual spatially separated fluorophores in each frame. The localization precision of the x-y coordinates is calculated at the work of Mortensen *et al*.^58^. We then compensated the sample drift by tracking the immobile fluorescent beads or by using cross-correlation. Only molecules localized in the range of 10–30 nm were displayed, with a fluorescent signal of more than 500 detected photons per frame. The distribution of the typical relevant parameters such as the precision of localization or the full width at half maximum of the point spread function are displayed in Fig. S3. The color intensity is directly proportional to the localization density per pixel. The *d*STORM images were reconstructed with a pixel size of 10 nm. The ZEN software outputs a text file containing a list of the x and y coordinates and the precision of all detected molecules in the time-series. We used this file to further visualize and treat the data with home-made Matlab program.

### Image Analysis

The reconstructed super-resolution image is filtered using a hysteresis threshold. The center of mass for each individual nuclear pore central channels is detected from the regional maxima of the H-maxima transform. NPC density is computed from the number of centers of mass weighted by area occupied by the nuclear envelope on the image. The NPC central channel diameter is measured from the reconstructed image by interpolation of the full width at half maximum of the radial autocorrelation. The NPC luminal ring diameter is determined by interpolation of the third zero-value of the radial autocorrelation computed derivative. Angular and radial density function is computed from the local sampling of neighbor couples in a distance window [d − 35 nm, d + 35 nm] from the considered center of mass and then averaged on all the centers of mass.

### Statistical test information

Alpha levels used in this work are 0.01. In other words we have considered the two tailed p-value tests significant for p < 0.01. The normality of our distributions has been tested by the evaluation of the first three moments of the distributions. We have used jack-knifing methods to assess that the standard error was a good estimate of the experimental error.

## Electronic supplementary material

Supplementary information
