# Supplementary material for: Nuclear pore complex plasticity during developmental process as revealed by super-resolution microscopy
Source: Sci Rep. 2017 Nov 7;7:14732. doi: 10.1038/s41598-017-15433-2 (PMC5677124; doi:10.1038/s41598-017-15433-2)

Nuclear pore complex plasticity during developmental process as revealed by super-resolution microscopy

Authors:

Julien Sellés[1], May Penrad-Mobayed[2], Cyndélia Guillaume[1], Alica Fuger[1], Loïc Auvray[1], Orestis Faklaris[3], Fabien Montel[1, 4*]

1 Matière et Systèmes Complexes, Université Paris Diderot/CNRS (UMR 7057), 75205 Paris Cedex 13, France

2 Institut Jacques Monod, Université Paris Diderot/CNRS, UMR 7592, 15 rue Hélène Brion, 75205 Paris CEDEX 13, France

3 ImagoSeine core facility, Institut Jacques Monod, Université Paris Diderot/CNRS, UMR 7592, 15 rue Hélène Brion, 75205 Paris CEDEX 13, France

4 Univ Lyon, Ens de Lyon, Univ Claude Bernard, CNRS, Laboratoire de Physique, F-69342 Lyon, France

* fabien.montel@ens-lyon.fr

Keywords: Nuclear pore complex, optical super-resolution microscopy, cell development, biophysics.

Supplementary information 1: Average image of the nuclear pore complex central channel. Pixel size: 10 nm. Image lateral size: 1.0 µm

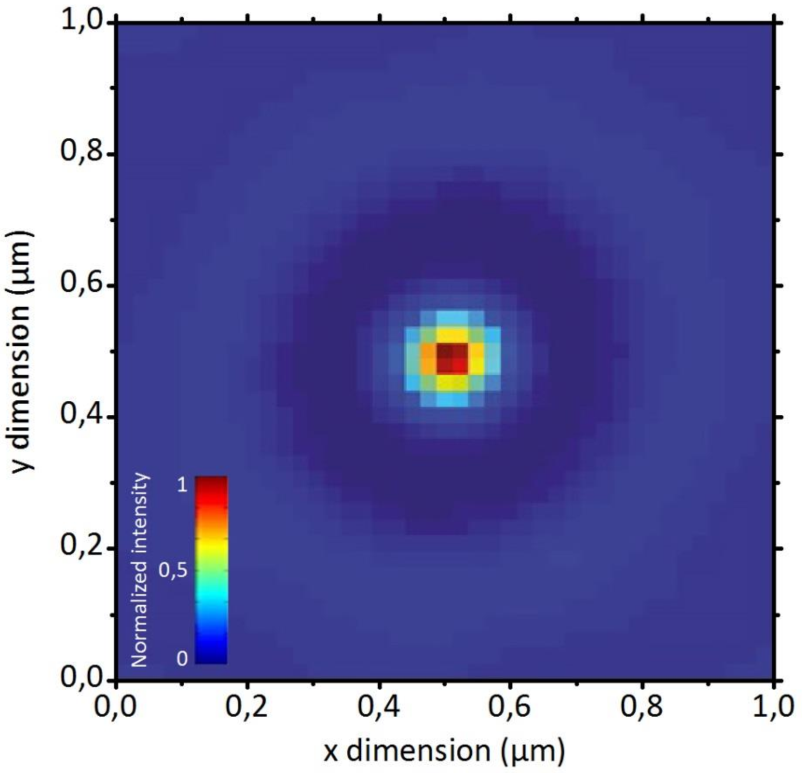

Supplementary information 2: Super-resolution images of NPCs central channel for different pixel size and different restrictions on the localization precision.

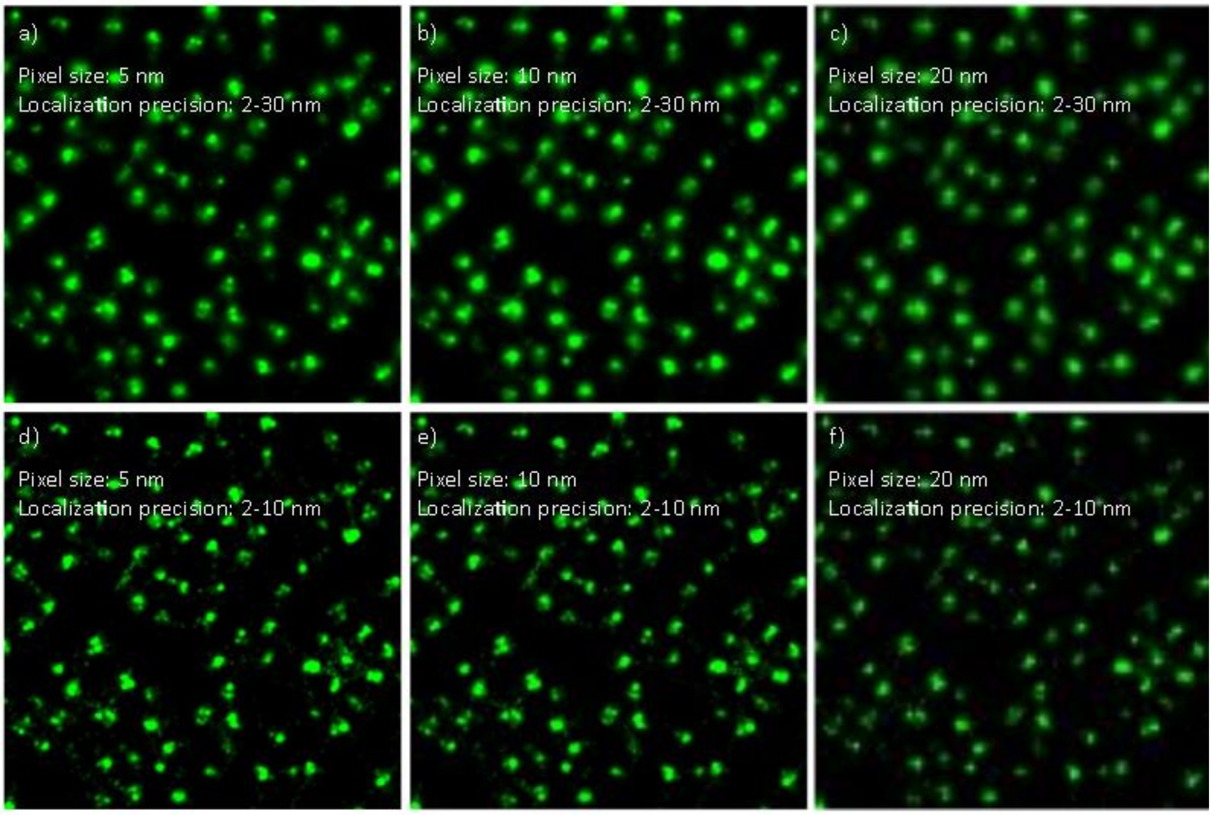

Supplementary information 2: Angular and radial density function P(d,α) for a) square lattice and for b) random soft disks 2D structures. First neighbor angle distribution evolution for c) a square lattice and d) a random lattice. N = 20 000 for each condition.

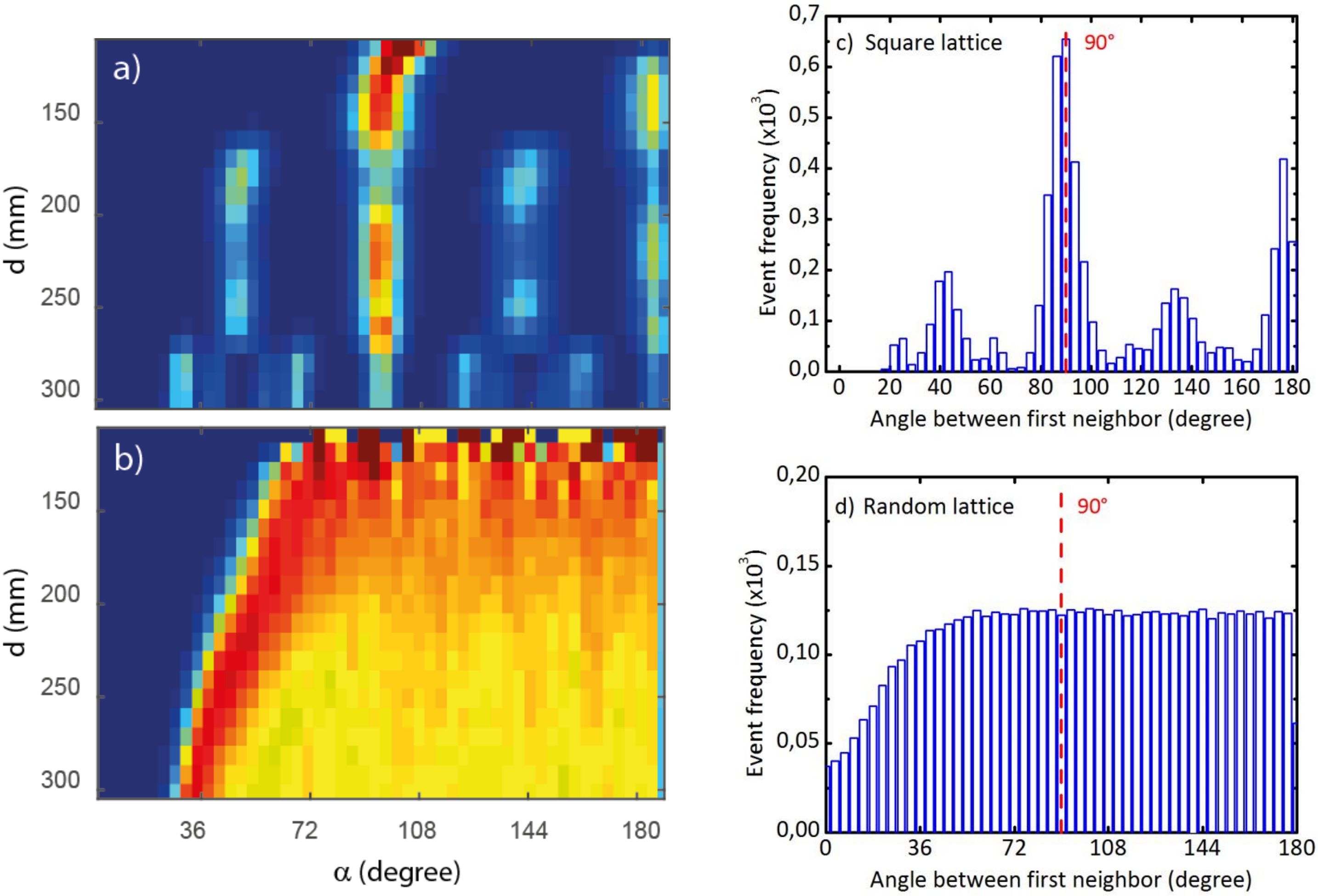

Supplementary information 3: Distribution of the typical relevant parameters and their boundaries (red dashed line) used for super-resolution reconstruction. a) Number of photons emitted per localization, b) Precision of localization per localization and c) Full width at half maximum of the point spread function per localization.

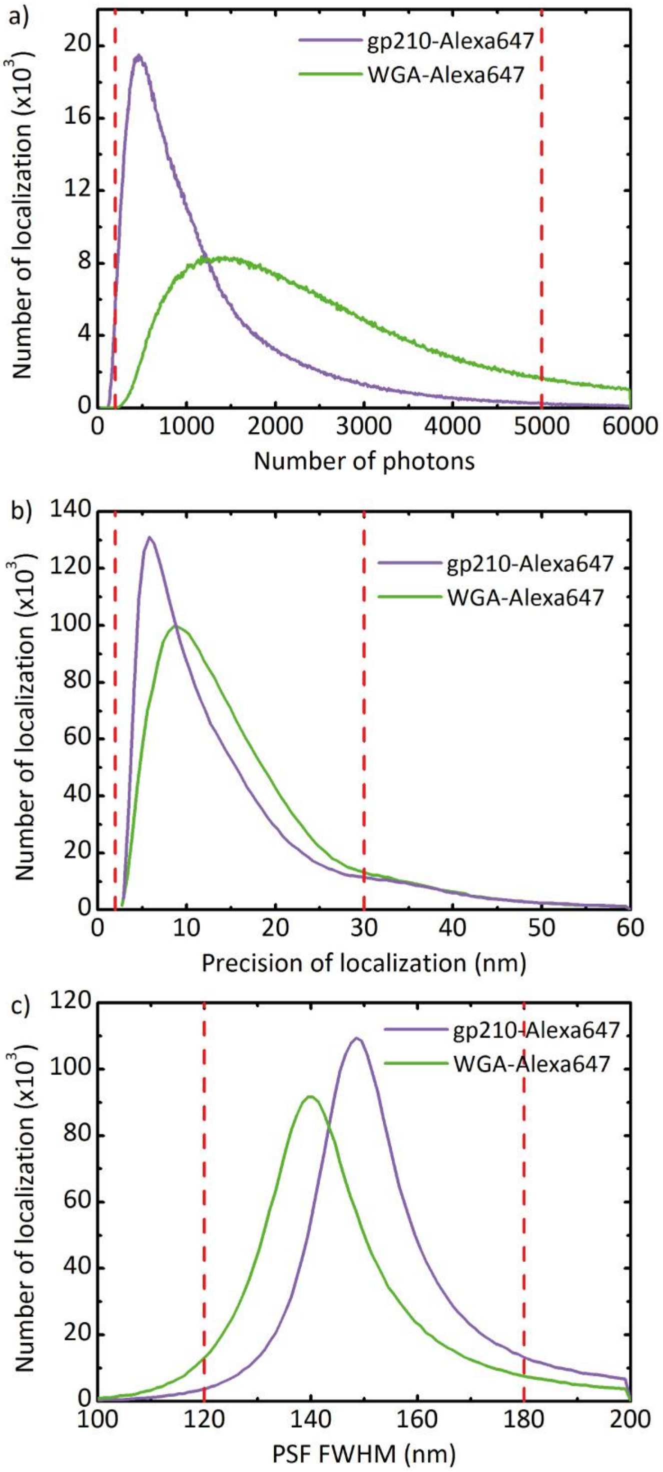

Supplement: Supplementary file 1 — Supplementary information [file 41598_2017_15433_MOESM1_ESM.pdf]
